# A Hospital-Based Cross-Sectional Study of Patients With Plantar Fasciitis: Is Hyperuricemia Screening Needed?

**DOI:** 10.7759/cureus.37088

**Published:** 2023-04-04

**Authors:** Shikha Yadav, Nitish Khandelwal, Saumen K Nath, Sanjay Rai

**Affiliations:** 1 Internal Medicine, Military Hospital, Ambala, IND; 2 Pathology, Military Hospital, Ambala, IND; 3 Radiology, Military Hospital, Meerut, IND; 4 Orthopaedics, Military Hospital, Ambala, IND

**Keywords:** foot pain, heel pain, screening, plantar fasciitis, asymptomatic hyperuricemia

## Abstract

Background and aim

Generally, asymptomatic hyperuricemia is considered a benign metabolic abnormality with little clinical significance in the absence of gout or renal calculus. However, its clinical association with plantar fasciitis is still not known and is a subject of interest. The study aims to investigate the association between asymptomatic hyperuricemia and plantar fasciitis in otherwise healthy patients.

Materials and methods

A cross-sectional study was performed, which included 284 patients aged 21-65 years with plantar fasciitis and without any comorbidities between February 2020 and November 2022. One hundred and fifty patients with hyperuricemia who attended the endocrinology and medicine outpatient department without heel pain were included as a control group. Serum uric acid levels were assessed in all cases. Student’s t-test, correlation tests, and multiple linear regression were used to ascertain the association between uric acid levels and plantar fasciitis. Statistical analyses were conducted using IBM SPSS Statistics for Windows, Version 19.0 (Released 2010; IBM Corp., Armonk, New York, United States).

Results

Among the 284 patients, 189 were female (66.5%) and 95 were male (33.4%). Their mean age was 43 ± 9 years (range: 21-65 years). The p-values of the duration of symptoms, visual analog scale for pain (VAS), and foot function index (FFI) total score were p = 0.061, p = 0.068, and p < 0.001, respectively. The mean uric acid levels were 7.6 ± 1.5 mg/dL in males and 7.3 ± 1.3 mg/dL in females in the sample group, and 8.3 ± 1.8 mg/dL in males and 8.1 ± 1.5 mg/dL in females in the control group. According to a Pearson correlation analysis, there was no correlation between serum uric acid level and BMI, VAS, duration of symptoms, FFI pain, disability sub-scores, or FFI total score.

Conclusion

Although asymptomatic hyperuricemia is a common metabolic abnormality, the present study did not find any significant association between it and plantar fasciitis. Therefore, we can conclude that routine screening for asymptomatic hyperuricemia is not recommended in plantar fasciitis. Evidence level: II

## Introduction

Plantar fasciitis affects millions of individuals globally. It is estimated that 7% of people over 65 years of age are diagnosed and treated for heel pain, which accounts for over one million physician consultations every year in the United States [1,2]. However, such data among Indian populations are not currently available.

Plantar fasciitis pain is usually at the anteromedial aspect of the heel. The intensity of the pain increases through dorsiflexion of the toes. On presentation, the patient may have had pain symptoms for weeks or months, which are often worse in the morning when trying to stand up from bed or walk. As soon as the patient starts walking, the intensity of pain generally reduces, but it can persist throughout the day and is often exacerbated by walking or exercise, especially on hard surfaces [3]. Many risk factors associated with plantar fasciitis have been studied, such as high physical activity, running, obesity in middle-aged (40-60 years) women [4-6], serving in the military [7-10], diabetes mellitus [11], tight Achilles tendon, pes cavus, pes planus [12], seronegative spondyloarthropathies, and gout [13]. Various seronegative spondyloarthropathies have been associated with plantar fasciitis, but in approximately 85% of cases, no known systemic abnormality is found [14,15].

Hyperuricemia is generally defined as a serum urate level above 7.0 mg/dL in men and above 6.0 mg/dL in women [16]. Asymptomatic hyperuricemia (AH) is defined as a serum uric acid level above 6 mg/dL without the occurrence of specific symptoms such as gout or renal stones [17]. There is increasing evidence that AH can be a risk factor for the development of obesity, hypertension, diabetes mellitus, and chronic kidney disease; however, its association with the development of plantar fasciitis has not been studied yet.

Therefore, the present study aimed to investigate whether plantar fasciitis is associated with AH in otherwise healthy, young individuals.

## Materials and methods

A cross-sectional study was performed, which included 284 patients aged 21-65 years with plantar fasciitis and without any comorbidities between February 2020 and November 2022. During the study period, 150 patients with other medical conditions like diabetes and hypertension who attended endocrinology and medicine OPD at Military Hospital, Ambala, Haryana, India, and were detected to have raised serum uric acid during the routine investigation but without any symptoms of plantar fasciitis were included as a control group.

Inclusion criteria were age between 21 and 65 years, no medical comorbidities, and not taking any medication that raises serum uric acid levels. The duration of the symptoms should be more than two weeks. All study-group patients were evaluated through rheumatological workup to rule out any existing inflammatory joint disease or autoimmune arthritis such as systemic lupus erythematosus (SLE), rheumatoid arthritis (RA), ankylosing spondylosis, psoriasis, and inflammatory bowel disease, which could cause plantar fasciitis or heel pain. The patients were also evaluated for calcaneal spurs by taking a lateral-view heel X-ray. BMI, job profile, and physical activity level were also recorded. The visual analog scale (VAS) was used to evaluate pain intensity, and the patients were asked to plot their scores. Informed written consent was obtained from all study participants. Approval from the Institutional Ethical Committee of Military Hospital, Ambala, India, was obtained (approval number: MHA/EC/Ortho/2020), and the study was conducted in accordance with the Helsinki Declaration.

Statistical analysis

We used IBM SPSS Statistics for Windows, Version 19.0 (Released 2010; IBM Corp., Armonk, New York, United States) and Microsoft Excel (Microsoft Corporation, Redmond, Washington, United States) for data analysis. We performed a power analysis to determine the appropriate sample size. A value of 0.05 was set for type I error (α), and the power of the test was set at 0.90 while maintaining a confidence level of 75.

Standard descriptive statistics were used to summarize the characteristics of the patients, including means and standard deviations (SDs) of all continuous variables and numbers, and percentages for categorical variables. We used Pearson’s correlation coefficient (r) to compare serum uric acid level against age, sex, BMI, symptom duration, morning stiffness, heel tenderness test positivity, foot function index (FFI) pain score, VAS, FFI disability score, FFI activity limitation score, and FFI total score. The statistical significance level was set at p<0.05.

## Results

A total of 295 patients with a main complaint of plantar heel pain were initially enrolled in our study. However, 284 patients were ultimately included in the study, as seven patients, upon clinical examination, were found to have radicular pain and lumbar spondylosis and were excluded. In addition, two patients were found to have ankylosing spondylitis, and two others had large calcaneal spurs as the cause of heel pain, and all four were also excluded.

In the study group of 284 participants, 189 were female (66.5%) and 95 male (33.4%). The mean age was 43 ± 9 years (range: 21-65 years). Right-side plantar fasciitis was noted in 78 patients (27.4%), left-side plantar fasciitis in 91 patients (32%), and bilateral plantar fasciitis in 115 patients (40.4%) (Table 1).

**Table 1:** Demographical characteristics

Parameters	Number of patients (n=284)	Control group (n= 150)	p-value
Gender			
Female	189 (66.5%)	87	0.051
Male	95 (33.4%)	63	
Mean Serum Uric Acid			
Female	7.3 ± 1.3 mg/dL	8.1 ± 1.5 mg/dL	0.001
Male	7.6 ± 1.5 mg/dL	8.3 ± 1.8 mg/dL	0.001
Employment Status			
Employed	108 (38%)	77	0.063
Unemployed	113 (39.7%)	14	
Retired	63 (22%)	60	
Foot Involvement			
Right	78 (27.4%)	-	0.062
Left	91 (32 %)	-	
Bilateral	115 (40.4%)	-	
Body Mass Index (kg/m^2^)			
Normal weight (18.5–24.9)	162 (57%)	79	0.043
Overweight (25–29.9)	101 (35.5%)	12	
Obesity (>30)	21 (7.3%)	59	
Level of Physical Activity			
Sedentary/office	19 (6.6%)	56	0.752
Standing work >6hr/day	69 (24.2%)	43	
Standing work <6hr/day	43 (15%)	35	
Walking >10 km/day	55 (19.3%)	9	
Walking <10 km/day	98 (34.5%)	7	
Duration of Symptoms			
2-6 weeks	103 (36.2%)	-	0.241
7-12 weeks	94 (33%)	-	
12-18 weeks	80 (28%)	-	
>18 weeks	7 (2.4%)	-	

The mean duration of symptoms was 8 ± 32 weeks (range: 4-112 weeks), and the mean VAS was 6 ± 1 (range: 3-10). Two hundred and forty-four patients (85.5%) had morning stiffness. The heel compression test was positive in 201 patients (70.7%), with plantar fascia tenderness present in all cases (Table 2).

**Table 2:** Pearson correlation analysis for various parameters and demographical parameters. VAS: visual analog scale; FFI: foot function index; BMI: body mass index

Parameter	Uric acid		FFI score		VAS	
	Correlation coefficient (r)	p-value	Correlation coefficient (r)	p-value	Correlation coefficient (r)	p-value
Age	0.367	0.087	0.208	0.013	0.119	0.326
VAS	0.389	0.068	0.778	<0.001		
Duration of Symptom	0.443	0.061	0.259	0.041	0.918	0.023
BMI	0.612	0.073	0.329	0.006	0.344	0.001
Morning Stiffness	0.742	0.061	0.114	0.314	0.255	0.029
FFI Pain Score	0.733	0.087	0.877	<0.001	0.873	<0.001
FFI Disability Score	0.711	0.082	0.891	<0.001	0.771	<0.001
FFI Activity limitation Score	0.314	0.066	0.285	0.018	0.103	0.266
FFI Total Score	0.467	0.0721			0.860	<0.001
Heel Compression Test	0.561	0.077	−0.452	0.699	−0.168	0.877

**Figure 1:**
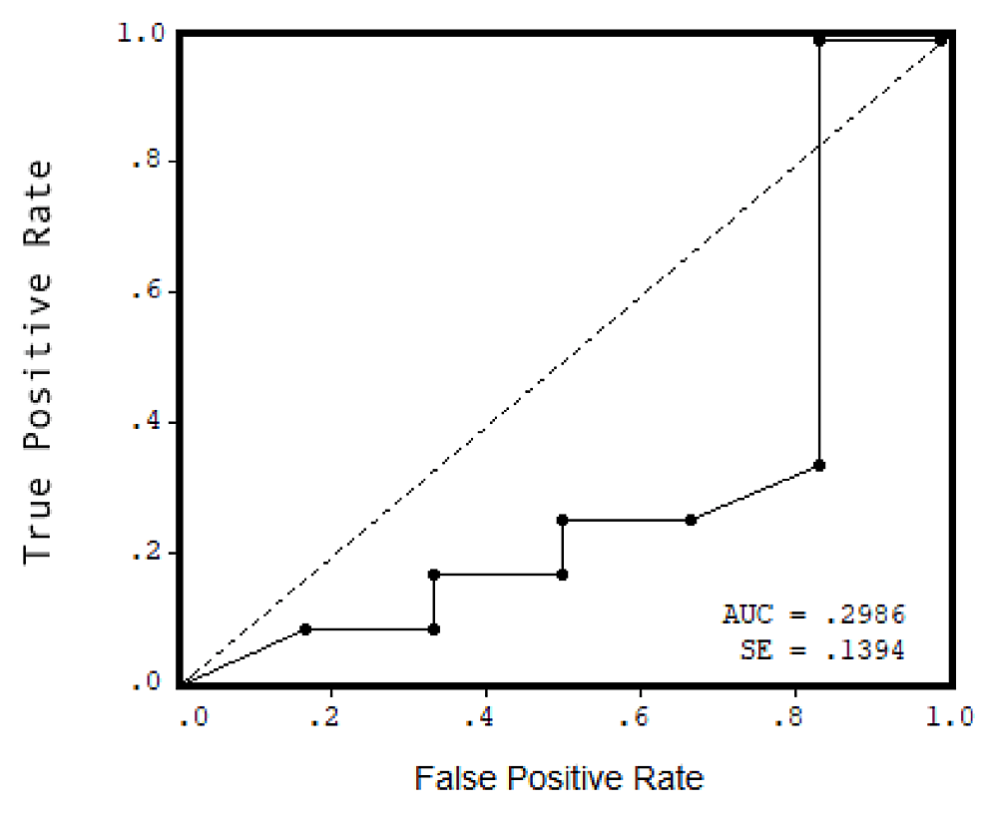
ROC curve showing non-association of asymptomatic hyperuricemia with plantar fasciitis ROC: receiver operating characteristic; AUC: area under the curve; SE: standard error

A Pearson correlation test was performed to ascertain the relationship between age, sex, serum uric acid level, BMI, duration of symptoms, presence of morning pain and stiffness, VAS, FFI pain, presence of heel compression test, FFI disability score, FFI activity limitation score, and FFI total score. However, no significant associations were found. After analyzing the receiver operating characteristic (ROC) curve, an area under the ROC curve (AUC) of 0.2986 (95%CI = 0.7016-0.876, p = 0.872) was observed between asymptomatic hyperuricemia and plantar fasciitis, indicating no association between them (Figure 1).

## Discussion

In this cross-sectional study, we attempted to evaluate the relationship between AH and plantar fasciitis. In our study population of otherwise healthy individuals attending orthopedic OPD due to foot pain, mean serum uric acid levels were within normal to hyperurecemic level in both males and females. A few authors have noted that in imaging studies, 25-40% of patients with AH have monosodium urate (MSU) crystal deposition [18,19]. The crystals are invariably deposited in joints and periarticular structures, especially at the first metatarsophalangeal joint, midfoot, and knee [20]. Pascual et al. showed that soft tissues like tendons and ligaments are often affected by MSU crystal deposition in gout patients [21].

Dalbeth et al. noted in their study of 92 gout patients that the Achilles tendon was affected in 39.1% of cases and peroneal tendons in 18.1% of cases, whereas other tendons were less or rarely involved [22]. The authors also noted that increased internal tissue stress during biomechanical loading does not play any significant role in patterns of MSU crystal deposition in gout [23]. Ames et al. noted that hyperuricemia can damage the microvascular structure and cause impaired tissue oxygenation, which in turn causes Achilles tendinopathy in gout patients [24]. However, no study has attempted to determine whether AH can cause plantar fasciitis, or whether plantar fasciitis and AH can co-exist. Therefore, the present study attempted to discover any association between AH and plantar fasciitis.

Comberg and Schach found that uric acid is associated with musculoskeletal joint pain [25]. Their results indicated the possible impact of urate level on unspecific joint pain, mainly affecting the lumbar spine, cervical spine, shoulder, and knee. Stewart et al., in their study of 24 patients with gout and 29 patients with AH, reported that those with AH who did not have any clinical features or symptoms of gout also complained of foot and leg pain [26]. A few studies have indicated that AH is associated with knee and foot pain, even in the absence of clinical gout [18,26]. Furthermore, uric acid levels were higher in patients having joint pain than in pain-free patients, gender-independent, and prevalent among patients above age 40. Similarly, Andersson and Leden, in a prospective study of 124 women, found a relationship between serum uric acid level and musculoskeletal pain, with a significant association between urate level and chronic non-gouty pain in women [27].

Gout can lead to significant morbidity by causing severe pain and limitation of activity through the deposition of urate crystals in the joints. In the present literature, it is well established that hyperuricemia plays an important role in the manifestation of gout, but whether it can also cause plantar fasciitis has not been studied thus far, nor whether AH alone is an indication to start anti-gout treatment. It is equally well established that the degree of hyperuricemia is a strong predictor in developing gout [26-28], but whether hyperuricemia also plays a role in the development of plantar fasciitis has not yet been studied. However, Nossent et al. noted that despite the high prevalence of hyperuricemia (10.7% of the population), uric acid was not independently predictive of cardiovascular disease or mortality. They further noted that hyperuricemia was a significant risk factor, associated with an increased cardiovascular death only in patients with gout and existing underlying cardiovascular disease [29]. Emerging evidence has suggested that hyperuricemia is an important risk factor for the development of atherosclerosis and cardiovascular disease [30-34]. Hsu et al. reported that in renal patients, hyperuricemia may be an independent predictor of musculoskeletal symptoms [35]. We did not find any study in the literature regarding the association of hyperuricemia with plantar fasciitis. Therefore, the present study may help future authors to further investigate and reach a definite conclusion.

Limitations of the study

The study has a few limitations. First, the patient-reported pain of plantar fasciitis is subjective and may lead to overdiagnosis. Second, the study was carried out in our hospital; therefore, hospital bias cannot be ruled out. Third, ultrasonographic imaging of the plantar fascia was not performed.

## Conclusions

Plantar fasciitis has many causes such as calcaneal spur, autoimmune disease, rheumatologic disorders, and ankylosing spondylosis. As isolated plantar fasciitis may not be associated with hyperuricemia, based on the present study, we do not recommend routine screening of patients with plantar fasciitis for AH, as both are not at all or poorly associated. However, the early detection of hyperuricemia in asymptomatic patients can give physicians the opportunity to modify or correct the underlying cause.
